# Intermediate metastatic risk lipomatous solitary fibrous tumor of the thigh (previously known as hemangiopericytoma): A rare case report with a literature review

**DOI:** 10.1097/MD.0000000000042052

**Published:** 2025-03-28

**Authors:** Ghefar Hmaydoosh, Youssef Ahmad, Muhsen Issa, Ghanem Ahmad, Duaa Knaj, Issa Ahmad, Rana Issa

**Affiliations:** a Faculty of Medicine, Tishreen University, Latakia, Syrian Arab Republic; b Faculty of Medicine, Al Andalus University for Medical Sciences, Tartus, Syrian Arab Republic; c Department of Vascular Surgery, Tishreen University Hospital, Latakia, Syrian Arab Republic; d Department of Oncology, Tishreen University Hospital, Latakia, Syrian Arab Republic; e Department of Pathology, Tishreen University Hospital, Latakia, Syrian Arab Republic.

**Keywords:** case report, intermediate risk, lipomatous hemangiopericytoma, lipomatous solitary fibrous tumor

## Abstract

**Rationale:**

Solitary fibrous tumor (SFT) is a rare mesenchymal tumor, which can arise at any anatomical location and exhibit a wide variety of histopathological characteristics. Lipomatous solitary fibrous tumor (LSFT) is a rare variant of SFT characterized by hemangiopericytoma-like vascular areas with a varying amount of adipocytic tissue. LSFT can be misdiagnosed with other tumors such as liposarcoma which can lead to unnecessary overtreatment for patients.

**Patient concerns:**

A 48-year-old man presented to the hospital clinic with a painless swelling located on the medial area of the upper thigh near the femoral artery, he has not seen any change in its size since he has noticed it a year ago.

**Diagnoses:**

Histological examination confirmed LSFT with an intermediate risk of metastasis.

**Interventions:**

Three-dimensional imaging technology with intravenous injection of contrast material was made and revealed a mass with well-vascularity supplied from the deep femoral artery. The lesion was excised and showed an intramuscular well-circumscribed encapsulated mass.

**Outcomes:**

The pathological findings LSFT with intermediate metastatic risk, and he is scheduled to receive radiotherapy for one and a half months. The majority of SFTs display benign histological characteristics and typically follow a slow clinical progression, leading to a favorable prognosis. To date, fewer than 20 cases of malignant LSFT have been documented. In our case, we utilized a recent tumor classification model that evaluates the metastatic potential of SFTs, and long-term follow-up is necessary because there is a chance of aggressive behavior.

**Lessons:**

LSFTs are usually found incidentally and often present as painless, movable masses. Even though they mostly follow a benign course, long-term follow-up is essential due to the possibility of turning malignant.

## 1. Introduction

Lipomatous solitary fibrous tumor (LSFT), formerly known as lipomatous hemangiopericytoma (LHPC), is a rare soft tissue tumor that poses diagnostic and therapeutic challenges due to its complex histopathological features. LSFT is classified as a rare variant of solitary fibrous tumors, intermediate (locally aggressive) (SFTs) under the World Health Organization classification in 2019.^[1]^

Usually, SFTs display a broad range of histopathological features, characterized by randomly arranged spindle to ovoid cells, thin-walled staghorn-shaped blood vessels, and collagen in the stroma. Variants such as fat-forming, giant cell-rich, and dedifferentiated forms are rare, but may also be encountered.^[2]^

SFTs can develop in individuals across a broad age spectrum, but they are uncommon in younger individuals and tend to occur in people in their 5th and 6th decades. The 1st case of SFT was identified in 1931 as a localized fibrous mesothelioma and since then, the tumor was thought to occur primarily in the lungs and pleura, however, a series of SFT cases outside the thoracic region was documented later in 1991. The pleura is the most commonly affected site, representing around 30% of cases, followed by the meninges (27%), abdominal cavity (20%), trunk (10%), extremities (8%), and head and neck (5%).^[3,4]^

Although the term LHPC was first documented by Nielsen et al in 1995 as a distinct entity, the 1st case in the literature is believed to have been reported in German by Theunissen et al in 1990.^[5,6]^ Until 1999, LHPC and SFT were considered separate tumors. In 2000, Guillou et al, by reporting 13 cases of LHPCs, noted that LHPC and SFT apart from the mature adipocytes share similar clinical, pathologic, immunohistochemical, and ultrastructural features, and suggested that lipomatous hemangiopericytoma represents a fat-containing variant of SFT.^[7]^

LSFT usually affects deep soft tissues but can also manifest rarely in various locations including the orbit, neck, parotid gland, mediastinum, stomach, retroperitoneum, and in our specific case, the thigh.^[3]^

This case report and literature review aim to provide a comprehensive overview of the current understanding of LSFT, showing its clinical presentation by reviewing previously reported cases.

## 2. Case presentation

**Figure 1.**
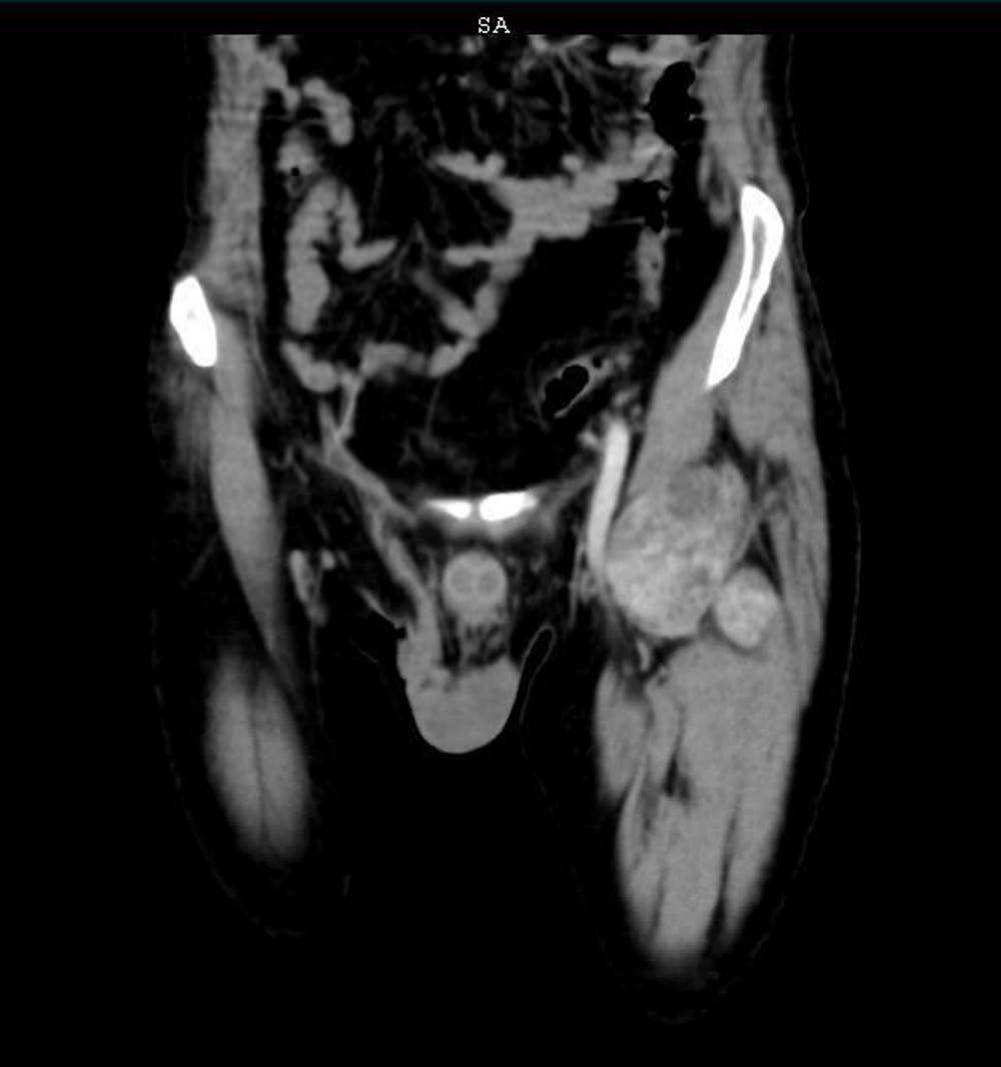
CT scan showing a well-defined mass in the top of the left thigh with well-defined margins. CT = computed tomography.

**Figure 2.**
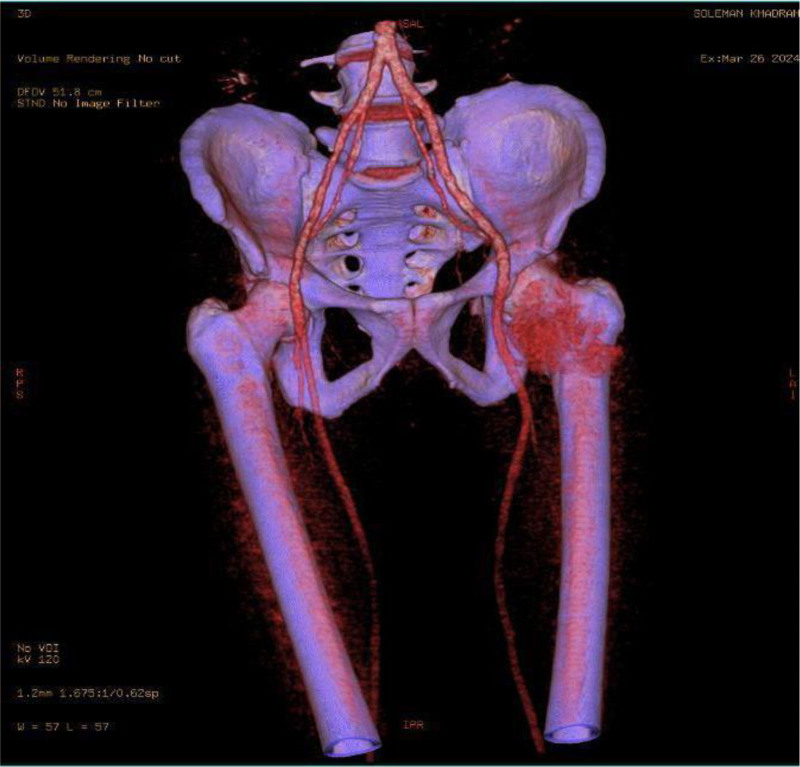
CT angiography revealing a highly vascularized tumor. CT = computed tomography.

**Figure 3.**
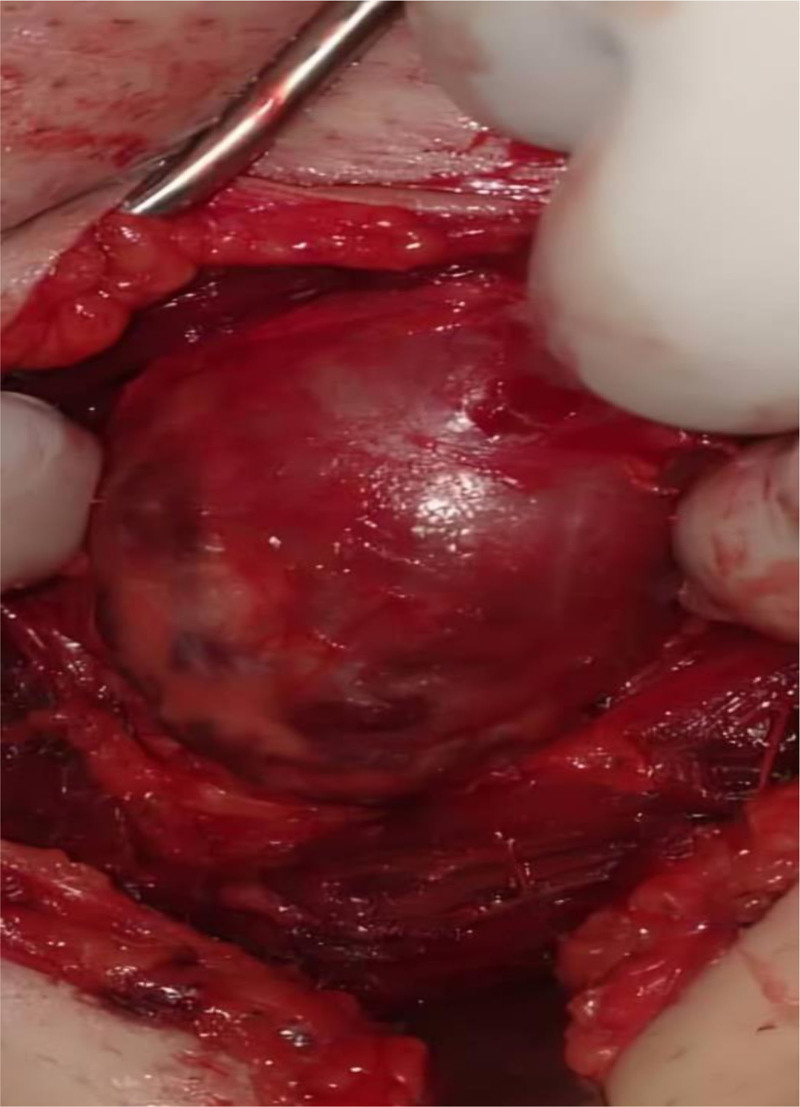
Surface of the tumor during the operation.

A 48-year-old male patient presented to the vascular clinic at Tishreen University Hospital with a painless swelling on the upper medial area of the thigh, near the femoral artery. He noticed it about a year ago, and reported no increase in its size since then. A physical exam showed a solid, movable mass under the skin that did not affect the pulse of the femoral artery or sensation in the area. The patient’s medical history is notable for hyperpituitarism, varicocelectomy 4 years ago, a right inguinal hernia operation in the same year, and paranasal sinus surgery in childhood. He is nonalcoholic and has been smoking hookahs for 20 years. He has no past allergies. A complete blood count, biochemical profile, and pituitary gland hormone tests were within normal range. A CT scan was done with very thin sections and a three-dimensional imaging technology focused on the left thigh arteries with an intravenous injection of contrast material (Fig. 1). The CT results showed a large well-vascularized mass in the root of the left thigh, lateral to the combined and superficial femoral arteries, with good vascular supply from the deep femoral artery (Fig. 2). The tumor measured (84 × 54) mm and extended along (93) mm without any connection to the superficial femoral artery. The high vascularity of the mass and its intramuscular location make sarcoma our first clinical differential diagnosis. After endocrine and cardiac consultations, the patient underwent surgery, where the whole tumor was completely excised and revealed an intramuscular well-circumscribed encapsulated mass with a smooth nodular surface (Fig. 3), attached focally to the upper part of the femoral quadriceps.

**Figure 4.**
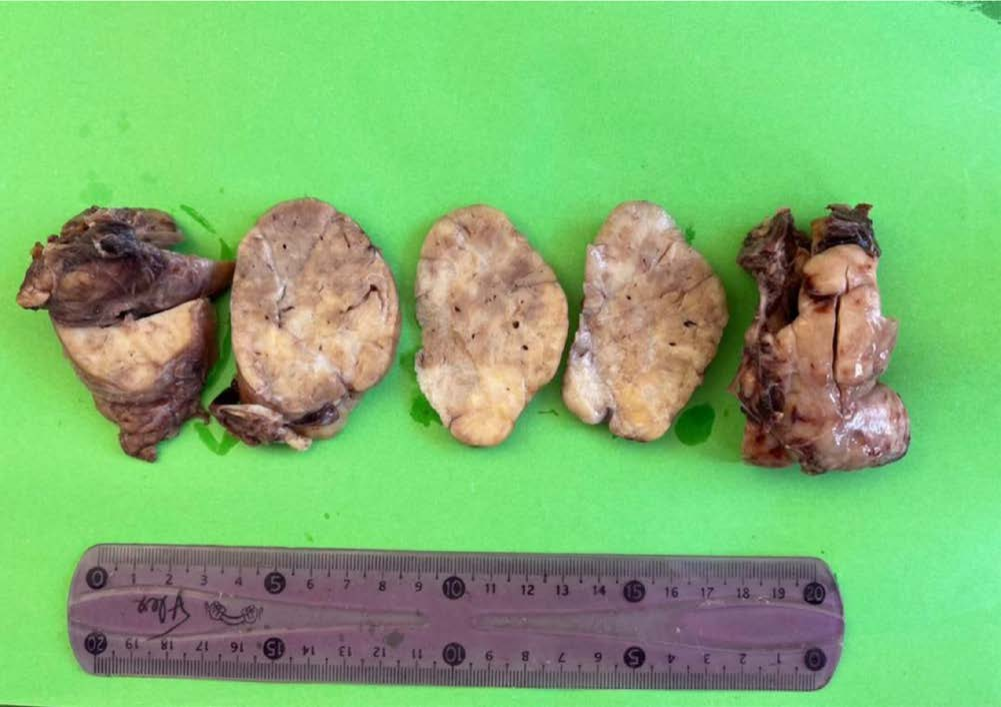
The cut section shows white gray and yellow cut surfaces, slit-like and hole-like spaces.

Grossly, the resected solid mass was encapsulated with relatively variegated cut sections, which were mainly gray-white with few light yellow areas. In addition, slit-like and hole-like spaces were seen (Fig. 4).

**Figure 5.**
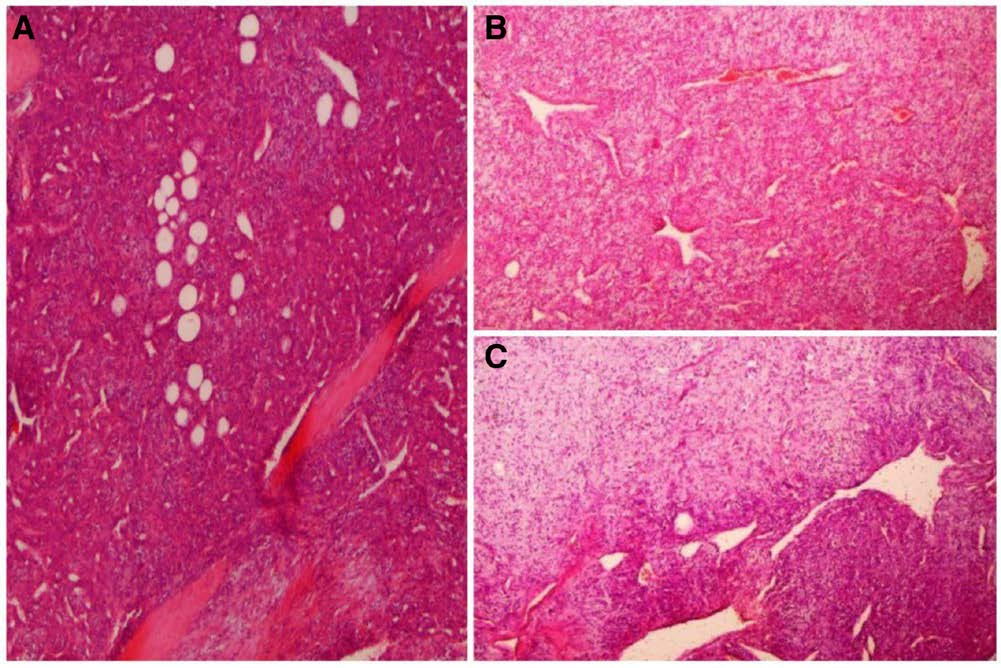
Branched vessels and mature fatty cells set in variably cellular collagenous stroma. Hematoxylin and eosin. (A) 40×, (B) 40×, (C) 40×.

**Figure 6.**
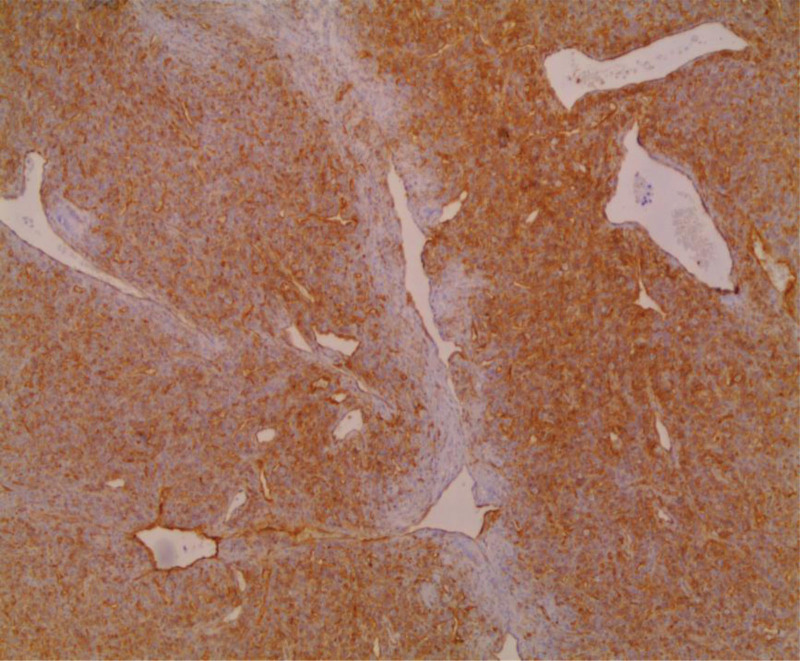
The tumor cells displaying strong positivity for CD34, 40×.

Microscopically, the tumor consisted of oval to spindle cells that were either randomly arranged or in short fascicles and had ill-defined cell borders. Staghorn-like ramified dilated vessels and mature lipocytes were intermixed among the tumor cells (Fig. 5). The collagenous stroma showed multiple foci of myxoid changes. In general, the cellularity of the tumor was variable, but there were many foci with high cellularity and increased mitotic activity, about 3–4 per 10 high-power fields. On the immunohistochemically stained sections, the tumor cells were positive for CD34 and negative for CD99, while lipocytes were positive for S-100 (Fig. 6). The pathology report indicated a lipomatous solitary fibrous tumor with intermediate metastatic risk (0 for the age +1 point for the tumor size + 2 points for mitotic activity = 3 points). After a 5-month follow-up, the patient remained in good health without any signs of recurrence. A written informed consent was obtained from the patient, and he is scheduled to receive radiotherapy for one and a half months.

## 3. Discussion

Fat-forming SFT or LSFT is a rare mesenchymal neoplasm that can occur at any age, usually developing in middle-aged individuals, between 40 and 60 years old. The youngest patient, 11 years old, was reported by Madala et al,^[8]^ while Lee et al reported the oldest patient, 93 years old.^[9]^ Articles have shown that extrapleural SFTs affect both genders equally, with a slightly higher occurrence in males for the fat-forming variant (male-to-female ratio 3:2).^[10]^ LSFT typically arises in deep soft tissue and has been reported in various sites. In our case, it was situated in the thigh, where only 14 other cases have been documented as shown in Table 1.^[7,9,11–15]^ Clinically, most documented cases of fat-forming SFTs exhibit morphological similarity to typical SFTs, except for varying quantities of mature lipocytes.^[7]^

**Table 1:** Clinicopathologic data for all reported cases of LSFT found in the thigh.

Reference	Sex/age	Location	Size (cm)
Ceballos et al 1999^[11]^	F/41	Thigh	9
Folpe et al 1999^[12]^	M/33	Thigh	6
	F/49	Thigh	13
	M/60	Thigh	21
	M/51	Thigh	17
Guillou et al 2000^[7]^	F/39	Thigh	10
Domanski et al 2003^[13]^	F/51	Thigh	5
Lee et al 2011^[9]^	M/55	Thigh	8.2
	M/78	Thigh	18
	M/45	Thigh	11.4
	F/20	Thigh	11
Noh et al 2014^[14]^	M/84	Thigh	11
Haller et al 2021^[15]^	M/49	Upper leg (thigh)	10.5
	M/57	Upper leg (thigh)	5
Our case	M/48	Thigh	8.4

F = female, LSFT = lipomatous solitary fibrous tumor, M = male, NA = not available.

SFTs are usually asymptomatic, slow-growing tumors that are often found incidentally on imaging. LSFTs appear as heterogeneous lesions on imaging, showing a mix of solid enhancing areas and low-density fatty regions on CT scans. This makes it difficult to distinguish them from other fat-containing tumors such as liposarcomas. The adipose areas exhibit strong signal intensity on T1-weighted magnetic resonance imaging, while the solid regions can have varying intensities. These regions may appear hypo, hyper, or isointense on T2-weighted images and hypo or isointense on T1-weighted sequences. During ultrasound examinations, LSFTs are heterogeneous and predominantly hyperechoic lesions with sound beam attenuation due to their fatty composition.^[16]^

LSFTs are slow-growing tumors often found fortuitously. Most SFT variants appear well-circumscribed on gross examination and may have a partial capsule with a nodular, white-cut surface. Some cases may show myxoid alterations and signs of bleeding.^[3]^ LSFTs are characterized by distinct histological features and often contain a mix of patternless cellular regions composed of round to spindle-shaped cells, hemangiopericytoma-like vascular areas, and a varying percentage of adipocytic areas.^[17]^

The discovery of a distinctive *NAB2–STAT6* fusion has shed light on the genetic basis of SFT. This fusion causes a constant activation of growth factors related to proliferation and survival, such as insulin-like growth factor 2 and fibroblast growth factor receptor 1. This genetic alteration is difficult to detect by conventional cytogenetics or fluorescence in situ hybridization testing. Therefore, immunohistochemistry plays a crucial role in diagnosing SFT. Detecting STAT6 by immunohistochemistry is a valuable indicator of fusions and is important for differentiating SFTs from similar tumors.^[2]^ Unfortunately, STAT6 was not available in our hospital. CD34 and Bcl-2 are also significant positive markers. In addition, LSFT generally shows strong positivity for vimentin and CD99, and occasionally, variable focal expression of epithelial membrane antigens such as S-100 and smooth muscle antibodies. Conversely, LSFT is usually negative for CD31, desmin, and cytokeratins.^[18]^

While most SFTs exhibit benign histologic features and have an indolent clinical course with a good prognosis, approximately 5%–10% of SFTs recur or metastasize, typically to lungs, liver, and bone.^[2,16]^ Traditional histologic parameters of SFT that indicate malignancy include pleomorphism, mitotic count >4/10 high-power fields, hemorrhage, and necrosis.^[16]^ A newer model in the latest World Health Organization Classification of tumors evaluates the metastatic potential of solitary fibrous tumors, which includes age, tumor size, and mitotic count. This model categorizes the patients into 3 classes (low risk with 0–2 points, intermediate with risk 3–4 points, and high risk with 5–6 points), and since our patient scored 3 points, he had an intermediate risk of metastasis.^[2]^

Distinguishing between benign and malignant SFTs based solely on imaging characteristics is not possible, due to limited data, as <20 malignant LSFT cases have been described in the English literature.^[14]^ In a 14 malignant case series, follow-up data from 10 cases (median duration of 47.5 months) showed 2 patients with multiple metastases. Although there is little evidence, metastasis is believed to be very rare in LSFTs.^[9]^

The prognosis of LSFT remains uncertain due to limited follow-up data in the literature, with only 30 cases followed up in English language literature and with suggestions that patients with LSFT should be followed up for more than 10 years.^[19]^ Long-term follow-up is planned in our case. Further research is required to investigate the biological behavior and prognosis of LSFT in order to determine the most effective treatment approach for this condition.

In the present case, the tumor was excised, and the patient is scheduled to receive radiotherapy to limit the risk of metastases and recurrence, however questions remains about the best treatment protocol and more evidence is needed in terms of long-term follow-up. The study has a limitation due to the absence of immunohistochemical validation of the STAT6 protein.

## 4. Conclusion

In this case report, a rare form of SFTs has been reported with a comprehensive literature review. According to our search in literature, there were only 14 previous cases of LSFT in the thigh. The substantial element is the malignancy of LSFT, which usually appears to have low proliferative activity and has little potential for local recurrence or metastatic spread in most cases. Long-term follow-up is crucial due to the possibility of behaving aggressively. Finally, to determine the most effective treatment and understanding of this rare tumor type more cases of LSFT should be studied and long durations of follow-up should be considered. This article seeks to enhance our understanding of these rare tumor variants and contribute to developing effective diagnostic and therapeutic approaches for patients with LSFT.

## Author contributions

**Conceptualization:** Ghefar Hmaydoosh, Issa Ahmad.

**Supervision:** Ghanem Ahmad, Issa Ahmad, Rana Issa.

**Resources:** Ghefar Hmaydoosh, Youssef Ahmad, Ghanem Ahmad, Duaa Knaj.

**Writing—original draft:** Ghefar Hmaydoosh, Youssef Ahmad, Issa Ahmad, Rana Issa.

**Writing—review & editing:** Ghefar Hmaydoosh, Youssef Ahmad, Muhsen Issa, Issa Ahmad, Rana Issa.
